# Tele-pulmonary rehabilitation with face to face in COVID-19 pandemic: A hybrid modeling

**DOI:** 10.5578/tt.20239908

**Published:** 2023-03-10

**Authors:** S. SATAR, M.E ŞAHİN, H. KARAMANLI, N. DEMİR, P. ERGÜN

**Affiliations:** 1 Clinic of Chronic Respiratory Failure, Pulmonary Rehabilitation Center, Ankara Atatürk Sanatorium Training and Research Hospital, University of Health Sciences, Ankara, Türkiye

**Keywords:** COVID-19, efficacy, hybrid model, ongoing symptoms, telepulmonary rehabilitation

## Abstract

**ABSTRACT:**

Tele-pulmonary rehabilitation with face to face in COVID-19 pandemic: A hybrid modeling

**Introduction:**

Post-illness pulmonary rehabilitation indications of Coronavirus disease-2019 (COVID-19) may include fatigue, respiratory restriction, exercise limitation, muscle weakness, deterioration in body composition, quality of life, and psychological status. Since tele-pulmonary rehabilitation (tele-PR) is the prominent approach in the current situation and questions such as who, how, and when are still unclear, in this study we aimed to investigate the efficacy of tele-PR as a hybrid model with face-to-face in post-COVID-19 patients.

**Materials and Methods:**

Thirty one patients who had completed viral infection treatment with the diagnosis of COVID-19 but still had persistent symptoms were enrolled in an eight-week synchronized video-conference mediated telePR program in a hybrid format, with the initial and final assessments and the first two sessions conducted in person. Before and after the tele-PR, pulmonary functions, exercise capacity, respiratory and peripheral muscle strength, body composition, quality of life, and psychological states were evaluated.

**Results:**

After the tele-PR program; a statistically significant improvement was observed in dyspnea sensation evaluated with modified Medical Research Council (mMRC) and BORG levels, body mass index (BMI), incremental shuttle walk test (ISWT), endurance shuttle walk test (ESWT), handgrip test, deltoid, and quadriceps 1-repetition maximum (1RM) results, maximal inspiratory and expiratory pressure (MIP, MEP), peripheral muscle strengths, fatigue severity scale and Nottingham extended activities of daily living scale (NEADLS).

**Conclusion:**

In this study, it has been shown that the hybrid model of tele-PR enables a comprehensive evaluation as well as the effective and safe applicability of a multidisciplinary and remotely directed program even in high workloads for post-COVID-19 patients.

## Abstract (tr)

**ÖZ:**

COVID-19 pandemisinde tele-pulmoner rehabilitasyon; yüz yüze ile hibrit model

**Giriş:**

Koronavirüs hastalığı-2019'un (COVID-19) hastalık sonrası pulmoner rehabilitasyon endikasyonları yorgunluk, solunum kısıtlaması, egzersiz kısıtlaması, kas zayıflığı, vücut kompozisyonunda bozulma, yaşam kalitesi ve psikolojik durumu içerebilir. Mevcut durumda tele-pulmoner rehabilitasyon (tele-PR) öne çıkan yaklaşım olduğu ve kim, nasıl, ne zaman gibi soruların hala belirsiz olduğu için, bu çalışmada COVID-19 sonrası hastalarda tele-PR’nin yüz yüze ile hibrit bir model olarak etkinliğini araştırmayı amaçladık

**Materyal ve Metod:**

COVID-19 tanısı ile viral enfeksiyon tedavisini tamamlayan ancak semptomları devam eden 31 hasta, ilk ve son değerlendirmeleri ve ilk iki seansı yüz yüze görüşme ile hibrit bir şekilde, sekiz haftalık senkronize video konferans aracılı tele-PR programına dahil edildi. Tele-PR öncesi ve sonrası solunum fonksiyonları, egzersiz kapasitesi, solunum ve periferik kas kuvveti, vücut kompozisyonu, yaşam kalitesi, psikolojik durumları değerlendirildi.

**Bulgular:**

Tele-PR programı sonrasında; Modifiye Medical Research Council (mMRC) ve BORG seviyeleri ile değerlendirilen dispne algısı, vücut kütle indeksi (BMI), artan hızda mekik yürüme testi (ISWT), dayanıklılık mekik yürüme testi (ESWT), el kavrama testi, deltoid ve kuadriseps 1-maksimum tekrar (1RM), maksimal inspiratuvar ve ekspiratuvar basınç (MIP, MEP), periferik kas kuvvetleri, yorgunluk şiddet ölçeği ve Nottingham genişletilmiş günlük yaşam aktiviteleri indeksi sonuçlarında istatistiksel olarak anlamlı bir iyileşme gözlendi.

**Sonuç:**

Bu çalışmada, tele-PR’nin hibrit modelinin, COVID-19 sonrası hastalar için multidisipliner ve uzaktan denetimli bir programın yüksek iş yüklerinde bile etkin ve güvenli uygulanabilirliğinin yanı sıra kapsamlı bir değerlendirmeye olanak sağladığı gösterilmiştir.

## Introduction

Pulmonary rehabilitation indications may differ as the course of COVID-19 disease changes on a scale ranging from mild to severe conditions such as acute respiratory distress syndrome (ARDS). Dyspnea and fatigue are the most common permanent symptoms after the active disease period ( 1 , 2 , 3 , 4 , 5 ). According to the guidelines, most patients who survive COVID-19 need physical, neuropsychological, and social support; and these patients, like other chronic respiratory disease patients, will benefit from pulmonary rehabilitation ( 6 ). During the pandemic, pulmonary rehabilitation facilities temporarily suspended operations, and telepulmonary rehabilitation, whether web-based, hospital-based, or at home, became a preferred concept ( 6 ). During the COVID-19 pandemic, our center was the first to use video-conference-mediated tele-PR as a hybrid method alongside the face-to-face format. Tele-PR is used in conjunction with face-toface rehabilitation to perform comprehensive initial and final evaluations using a multidisciplinary approach, as well as to determine the necessity for careful monitoring throughout sessions. This study aimed to evaluate the effectiveness of a hybrid model of tele-PR in post-COVID-19 patients.

## MATERIALS and METHODS

### Study Population

Patients who have been diagnosed with COVID-19 and still had active symptoms were referred to our pulmonary rehabilitation unit. During this period, a total of 72 post-COVID-19 patients received PR. Two patients did not attend the PR program, 39 of them were included in the unsupervised home-based exercise program with telehealth support, while 31 patients were included in the supervised home-based exercise program via telerehabilitation. Although new patients are being included in the program, we analyzed the data of our first 31 patients who completed the multidisciplinary comprehensive supervised hybrid tele-pulmonary rehabilitation with the face-to-face method. Written and signed informed consent forms were obtained from the patients before the study onset.

Before starting the study, the G-power analysis was performed, and it was determined that a minimum of 25 patients with a confidence interval of 0.80 and a margin of error of 0.05 should be included in the study.

#### Inclusion Criteria

• Patients over the age of 18, • Patients diagnosed with polymerase chain reaction (PCR) (+) COVID-19 and/or had highresolution computed tomography (HRCT) imaging compatible with COVID-19, • Patients who had COVID-19 confirmed 4-6 weeks before the study using the procedures described above, • Patients with intensive care unit (ICU) hospitalization and/or intubation history due to COVID-19, • Patients with a prolonged hospitalization without ICU admission or high-flow oxygen/non-invasive mechanical ventilator (NIMV) therapy, • Patients who still had modified Medical Research Council dyspnea (mMRC) scores ≥2 at 4-6 weeks after diagnosis without hospitalization, • Patients who had symptoms that impair the quality of life after COVID-19 in the post-acute period and stable state, • Patients who could access technological devices with a real-time video camera function (smartphone, tablet, laptop, desktop computer, or smart television) with their home internet connection were included in the study.

#### Exclusion Criteria

• Patients who tested positive for active coronavirus infection 24 to 72 hours before the start of the program, • Patients with a fever of ≥38 degrees Celsius, • Patients included in inpatient PR practice due to their severe symptoms, • Patients with a diagnosis of COVID-19 who required a specific rehabilitation program, • Patients who were diagnosed with COVID-19 but also diagnosed with concurrent cancer and were still undergoing cancer treatment, • Patients with uncontrolled hypertension, unstable angina pectoris or myocarditis, active pulmonary thromboembolism, • Patients who had a high fall risk but did not have family support, • Patients who could not complete the program or whose full results were not obtained were excluded from the study.

#### Ethical Consideration

Institutional review board approval was obtained on Jan 12, 2021, with the ethics committee approval number 2012-KAEK-15/2213 after receiving approval from our own hospital’s medical specialization education board (dated December 3, 2020, and number 703-5) that the study can be conducted therein.

#### Outcome Measures

The initial and final evaluations of our patients were made face-to-face in our PR center under personal protective precautions. The Modified Medical Research Council Dyspnea (mMRC) and modified BORG (mBORG) scale values of the patients were questioned and recorded for the perception of dyspnea ( 7 , 8 ). Exercise capacity was evaluated using the incremental shuttle walking test (ISWT) and endurance shuttle walking test (ESWT), the tests were performed according to field walking tests guidelines ( 9 , 10 ). Respiratory muscle strength was measured using a Micro-RPM respiratory pressure meter (CareFusion, Hoechberg, Germany) and the maximal inspiratory pressure (MIP) and maximal expiratory pressure (MEP) values were recorded. MIP and MEP were measured by the same physiotherapist while the patient was in a sitting position, following the recommendations of the American Thoracic Society and European Respiratory Society (ATS-ERS) starting from residual volume and total lung capacity, respectively ( 11 ). The best value was recorded after the tests were repeated a minimum of three times. To assess the peripheral muscle strength of the patients, a handgrip test was performed using a hand dynamometer. Also, a 1-repetition maximum (1-RM) test is performed to evaluate peripheral muscle strength. Bioelectrical impedance was used to assess the body compositions of the patients using a TANITA device (TBF-300A Total Body Composition Analyzer, Tokyo, Japan). Body mass index (BMI) and fat-free mass index (FFMI) were calculated using the formula of weight (body mass for BMI, fat-free mass for FFMI) in kilograms divided by the square of the height in meters. Finally, the Nottingham extended activities of daily living scale (NEADLS) was used to objectively measure the affected activities of daily living and disability levels of post-COVID-19 patients. Also, the hospital anxiety-depression score was used for the psychological conditions of the patients, and the fatigue severity scale was used for the fatigue status of the patients.

#### Pulmonary Rehabilitation Program

In this study, the tele-PR program was applied in a hybrid manner with the face-to-face format in 17 patients who were referred after having COVID-19. The initial and final evaluations, as well as the first two sessions, were conducted face-to-face at our center while taking personal protective measures. Patients who applied for PR were asked to confirm that they had a negative COVID-19 PCR test result 24-72 hours prior to the first evaluation. The cardiac status of all patients was checked with echocardiography at baseline. Following a thorough medical history and physical examination by a chest physician, patients were examined by a physiotherapist, psychologist, nutritionist, and nurse as part of a multidisciplinary team. Programs were tailored to individual needs, ability to tolerate the exercise and disease severity. The PR program consisted of exercise training, education, and nutritional and psychosocial counseling. The first two sessions were performed face-to-face at the hospital. During these sessions, training on the nature of the disease, self-management strategies, the importance of exercise training, breathing training, bronchial hygiene techniques, energy conservation techniques, medication advice, dietary advice, and psychosocial support was also provided. The patients then enrolled in an eight-week supervised home-based synchronized tele-PR program using video conferencing. The program was implemented two days a week. The heart rate, blood pressure, and oxygen saturation of the patients were monitored online during the PR sessions. When necessary, oxygen support was provided to the patients to keep the oxygen saturation above 90%. The exercise training program consisted of endurance and resistance training. The endurance training included 30 minutes (min) of endurance exercise at 85% of each patient’s VO2 peak calculated from the ISWT. The length of the corridor at the patients’ home was determined and a walking program was created according to the number of tours that should be completed within 30 minutes. The program was adjusted for those who had a treadmill at home. A 15-minute warm-up and cool-down period were also included. For strengthening exercises, a program consisting of two sets of 12-18 repetitions was applied in 30-50% of a maximum repetition using free weights provided by the patients. The training was based on the recommendations of the guidelines ( 11 , 12 ). If necessary, inspiratory muscle training (IMT) was performed twice a day with an inspiratory load of 30-50 percent maximum inspiratory pressure for 5-10 minutes.

#### Statistical Analysis

Statistical analyses were performed by IBM SPSS version 26.0 (IBM SPSS Statistics for Windows, Chicago, IL, USA). Normally-distributed numeric variables were expressed as mean and standard deviation while non-normally distributed variables were expressed as median and interquartile range. Categorical variables were expressed as numbers and percentages. To determine if the variables were normally distributed, visual (histograms, probability plots) and analytical methods (Shapiro-Wilk, skewness, and kurtosis test) were used. When comparing PR parameters before and after the PR program, the Wilcoxon test was employed if the data were not normally distributed, and the paired t-test was used if they were. A p-value of 0.05 was used to determine statistical significance.

### RESULTS

The data of the first 31 patients who completed the rehabilitation program was analyzed. The mean age of our patients was 57.35 ± 9.47 years. Twenty-four (77.4%) were male and seven patients (22.6%) were female. The mean time from diagnosis of COVID-19 to the onset of PR was 86.70 ± 67.36 days. While shortness of breath was the most common symptom reported by our patients, additional symptoms included weakness, cough, and fever. Eleven (35.4%) of our patients were non-smokers, the remaining 20 (64.5%) were ex-smokers and the average cigarette consumption was 27.75 ± 19.85 pack years. Four of our patients had COPD, three had asthma, seven had diabetes mellitus, and six had hypertension as a comorbidity. While 23 of our patients received COVID-19 treatment in the hospital (average length of stay: 19.95 days), eight of them had to be followed in the intensive care unit during their hospitalization. While the minimum length of ICU stay of these eight patients was four days, the maximum was 50 days, and the average length of ICU stay was 16.88 days. All detailed demographic data and initial evaluation parameters of the patients are given in Table 1.

A statistically significant improvement was found in the assessment of dyspnea perception performed by mMRC after tele-PR (p< 0.001). There was a statistically significant improvement in the BORG value measured at rest and after exercise (p= 0.008 and p< 0.001, respectively). A statistically significant increase was found in exercise capacity, which was evaluated by ISWT and ESWT, after the program (p< 0.001 and p< 0.001, respectively). MIP and MEP values used in the measurement of respiratory muscle strength also increased after PR, and the increases were found to be

**Table 1:** Patient data at first evaluation

	Mean ± SD n (%)	Median percentiles	Median percentiles	Median percentiles
		25	50	75
Gender (m/f) n (%)	24 (77.40%)/7 (22.60%)	-	-	-
Age (years)	57.35 ± 9.47	49.00	57.00	63.00
Smoking (p/year)*	27.75 ± 19.85	6.25	30.00	48.75
Time from diagnosis to onset of PR (days)	86.70 ± 67.36	56.00	71.00	97.00
Hospitalization days*	19.95 ± 17.57	7.00	15.00	22.00
ICU days*	16.88 ± 14.92	6.00	13.00	22.25
BMI (kg/m2)	28.21 ± 4.00	26.60	27.60	29.60
FFMI (kg/m2)	55.49 ± 7.90	51.00	56.60	60.45
mMRC score*	2.32 ± 0.87	2.00	2.00	3.00
Hospital anxiety score*	5.48 ± 2.65	3.00	6.00	7.00
Hospital depression score*	6.79 ± 2.74	4.50	6.00	10.00
BORG at rest*	0.32 ± 0.59	0.00	0.00	1.00
BORG after exercise*	3.58 ± 1.02	3.00	4.00	4.00
ISWT (meters)*	348.15 ± 124.09	270.00	370.00	430.00
ESWT (min)*	9.99 ± 6.34	5.35	8.20	13.38
MIP (cmH2O)	101.83 ± 27.53	85.75	100.50	121.50
MEP (cmH2O)	123.56 ± 27.99	103.50	124.50	147.75
Handgrip test	33.27 ± 10.02	26.00	30.00	40.50
Deltoid 1-repetition maximum (kg)*	5.17 ± 1.58	3.75	5.00	7.00
Quadriceps 1-repetition maximum (kg)*	11.44 ± 4.31	7.75	10.00	15.00
NEADLS	51.48 ± 15.55	43.00	58.00	64.50
Fatigue severity scale	4.91 ± 1.63	3.71	5.44	6.20

*: The non-normally distributed data specified with *. BMI: Body mass index, ESWT: Endurance shuttle walking test, FFMI: Fat-free mass index, ICU: Intensive care unit, ISWT: Incremental shuttle walking test, LTOT: Long-term oxygen treatment, m/f: Male/female, MEP: Maximal expiratory pressure, min: Minute, MIP: Maximal inspiratory pressure, mMRC: Modified medical research council, n: Number, NEADLS: Nottingham extended activities of daily living scale, p/year: Packet/year.

statistically significant (p= 0.014 and p< 0.001, respectively). In both the right and left handgrip test, deltoid and quadriceps 1-repetition maximum values, which were checked for peripheral muscle strength, a statistically significant increase in strength was observed in all of them. In terms of body composition, there was a statistically significant increase in BMI value after the program. Furthermore, according to the results of the Nottingham extended activities of daily living scale, which is one of the indicators of disability, and the fatigue severity scale used for fatigue assessment, the patients’ activities of daily living and fatigue levels improved statistically significantly (p= 0.001 and p< 0.001, respectively). Results and statistical comparison of all parameters checked before and after PR are given in Table 2.

### DISCUSSION

This publication has presented the results of the first video-conference-mediated tele-PR program in a hybrid setting with the face-to-face method in patients with COVID-19 during the pandemic in Türkiye. It was shown that this model positively affects the dyspnea perception, exercise capacity, muscle strength, body composition, and daily living activities of patients with ongoing symptoms after COVID-19.

As noted in recent publications, the most common ongoing symptoms after COVID-19 were fatigue, dyspnea, neuropsychological problems, and reduced quality of life ( 1 , 2 , 4 ). It is also known that these symptoms may persist for a long time after the illness.

**Table 2:** Demographic variables, comorbidities, management and outcomes of VAP survivors and non-survivors

	Mean ± SD (%)	Median percentiles (interquartile range) 50 (25: 75)	Mean ± SD(%)	Median percentiles (interquartile range) 50 (25: 75)	p
mMRC score*	2.32 ± 0.87	2.00 (2.00: 3.00)	1.39 ± 0.71	2.00 (2.00: 3.00)	<0.001
BORG at rest*	0.33 ± 0.60	0.00 (0.00: 1.00)	0.00 ± 0.00	0.00 (0.00: 0.00)	0.008
BORG after exercise*	3.53 ± 1.00	4.00 (3.00: 4.00)	2.57 ± 1.04	2.50 (2.00: 3.00)	<0.001
ISWT (meters) *	351.15 ± 125.54	370.00 (270.00: 430.00)	445.38 ± 106.51	450.00 (350.00: 540.00)	<0.001
ESWT (min) *	10.29 ± 6.27	8.20 (5.35: 13.38)	15.94 ± 5.24	20.00 (13.00: 20.00)	<0.001
MIP (cmH2O)	102.82 ± 28.04	100.50 (85.75: 121.50)	112.88 ± 33.52	111.00 (82.75: 125.00)	0.014
MEP (cmH2O)	124.12 ± 28.75	124.50 (103.50: 147.75)	143.71 ± 35.96	135.00 (114.00: 162.25)	<0.001
Hand grip test-Right	33.07 ± 10.13	30.00 (26.00: 40.50)	36.64 ± 9.63	34.00 (30.00: 42.00)	<0.001
Hand grip test-Left	31.86 ± 10.15	31.00 (24.00: 42.00)	34.21 ± 9.35	34.00 (30.50: 41.00)	<0.001
Deltoid 1RM (kg) *	5.17 ± 1.58	5.00 (3.75: 7.00)	6.44 ± 2.72	5.00 (4.00: 9.00)	0.002
Quadriceps 1RM (kg)*	11.53 ± 4.43	10.00 (7.75: 15.00)	13.76 ± 4.61	12.00 (10.00: 17.00)	0.001
BMI (kg/m2)	28.21 ± 4.00	27.60 (26.60: 29.60)	28.95 ± 3.69	29.00 (27.20: 30.30)	0.002
FFMI (kg/m2)	19.55 ± 1.76	20.10 (18.70: 20.75)	19.83 ± 2.01	20.40 (18.10: 21.40)	0.079
NEADLS	51.48 ± 15.55	58.00 (43.00: 64.50)	59.48 ± 11.11	65.00 (59.00: 66.00)	0.001
HADa*	5.48 ± 2.65	6.00 (3.00: 7.00)	5.59 ± 2.79	6.00 (3.00: 7.00)	0.948
HADd*	6.79 ± 2.74	6.00 (4.50: 10.00)	5.79 ± 3.21	6.00 (3.00: 9.00)	0.314
Fatigue severity scale	4.91 ± 1.63	5.44 (3.71: 6.20)	3.68 ± 1.52	4.00 (2.33: 5.11)	<0.001

*: The non-normally distributed data specified with. Statistically significant data (p< 0.05) were indicated in bold. 1RM: 1-repetition maximum, BMI: Body mass index, ESWT: Endurance shuttle walking test, FFMI: Fat-free mass index, HADa: Hospital anxiety score, HADd: Hospital depression score, ISWT: Incremental shuttle walking test, MEP: Maximal expiratory pressure, min: Minimum, MIP: Maximal inspiratory pressure, mMRC: Modified medical research council, NEADLS: Nottingham extended activities of daily living scale, SD: Standard deviation, tele-PR: Tele-pulmonary rehabilitation.

In a study using data from 152 patients who survived COVID-19, approximately three-quarters of the patients showed persistent shortness of breath even 7-9 weeks after the onset of the disease ( 5 ). In another study, it was shown that patients who were hospitalized for COVID-19 had fatigue and dyspnea even 110 days after discharge ( 4 ). Even after 4-6 weeks from diagnosis, the most common symptoms in our patients at admission to our PR center were shortness of breath, exhaustion, and cough, with twelve of them having a history of hospitalization.

Providing pulmonary rehabilitation via telehealth during COVID-19 is the most prominent model, but questions such as “to whom, how, and when?” remain unanswered.

It has been demonstrated that, as far as is known, telerehabilitation is most routinely employed in stroke patients ( 13 ). In the field of COPD, several publications suggest that tele-PR is equally effective as conventional hospital-based PR ( 14 , 15 ). However, there are very few publications and data on tele-PR in COVID-19 patients. Zhao et al. ( 16 ) recommended that pulmonary rehabilitation should be provided to patients diagnosed with COVID-19 in isolation or in the community through instructional videos, instruction manuals, or telerehabilitation if necessary. In an article by Salawu et al. ( 17 ), it is recommended that each patient with a diagnosis of COVID-19 discharged from the hospital should be referred to appropriate centers for tele-PR in case of residual deficit. According to Vitacca M. ( 18 ), improvements in exercise tolerance, dyspnea, and muscle fatigue were obtained with an 88 percent compliance to a 30-day telerehabilitation program in a subgroup of post-COVID-19 patients with a low exercise capacity, exercise-induced desaturation, mildly restrictive ventilation pattern, and persistent pathological lung imaging.

It’s a known fact that the pulmonary rehabilitation program in COVID-19 should be comprehensive, covering not only cardiorespiratory and motor dysfunction but also the consequences of neurological deterioration, and worsening of comorbidities ( 19 , 20 ). There is no clear information about the content of PR that should be applied in COVID-19 cases. In a study, PR was recommended for all mild, moderate, and severe post-COVID patient groups at very low workloads ( 16 ). In a case series published by Wootton et al. ( 21 ), three patients were evaluated remotely using a five-repetition and one-minute sitto-stand test, and they were exercised at low workloads with BORG levels below three for six weeks with a multidisciplinary team coordinated by a physiotherapist. Mukaino et al. ( 22 ) used telerehabilitation on four patients with a history of hospitalization, with each session lasting 20 minutes. At the end of the program, all patients gave positive feedback about the program’s efficiency.

In our study, the initial and final evaluations and the first two sessions were performed face-to-face. Thus, the hybrid approach not only allowed the cases to be evaluated comprehensively but also allowed the cases to be monitored concurrently via video calls during the sessions, which is the method’s strength. Unlike previous studies, the patients in our PR practice had an eight week program and were maintained at higher workloads calculated from ISWT. With this approach, endurance exercises could be performed at high workloads such as 60-80% of VO2 peak.

Not only the reduced exercise capacity but also the deterioration of body composition is an important problem in COVID-19.

Vomiting, anorexia, and reduced food intake are observed in 25.8% of all symptomatic COVID-19 patients, with a higher incidence in critical patients ( 23 ). The anxiety of disease, isolation stress, lack of social interaction during illness, dyspnea, dysosmia, and dysgeusia can be considered among the reasons for this malnutrition in COVID-19, especially in older patients ( 24 ). Even though the majority of our patients were overweight prior to PR, a statistically significant increase in BMI was observed, which may be associated with improvements in physical performance, quality of life, and psychological status.

Fear, anxiety, depression, and post-traumatic stress symptoms have all been reported as a result of the COVID-19 pandemic, which has many unknowns ( 25 ). Therefore, the psychological support needs of the patients should also be taken into consideration in PR programs. But the fact that our video conferencing-mediated sessions were held simultaneously with multiple patients, as well as the lack of individual psychological counseling, may explain the failure to achieve the desired improvement in anxiety-depression levels. After eighteen sessions of tele-pulmonary rehabilitation even with the increased anxiety levels, there were improvements in activities of daily living and COVID-19-related fatigue levels.

### Limitations

The limitation of this study is that it includes the results of a single center and a small number of patients. Although the absence of a control group is also seen as a limitation, the study was carried out on a single arm, since hospital-based face-to-face direct supervised PR could not be performed on a similar group of patients due to the risk of transmission during the pandemic. Another disadvantage is that despite the comprehensive evaluation, telepsychological counseling could not be provided within the scope of telehealth due to the inadequate facilities of our center.

### CONCLUSION

This study demonstrated that a hybrid model of telePR and face-to-face sessions allows for a thorough evaluation as well as the effective and safe use of a multidisciplinary and comprehensive program.

#### Ethical Committee Approval:

This study was approved by Ankara Keçiören Training and Research Hospital Clinical Research Ethics Committee (Decision no: 2012-KAEK-1512213, Date: 12.01.2021).

#### Conflict of Interest

The authors declare that they have no conflict of interest.

### AUTHORSHIP CONTRIBUTIONS

Concept/Design: SS, MEŞ, PE

Analysis/Interpretation: All of authors

Data acqusition: All of authors

Writing: SS, MEŞ, PE

Clinical Revision: SS, MEŞ, PE

Final Approval: SS, MEŞ, PE
